# The role of life‐course socioeconomic position in cognitive change and mild cognitive impairment among middle‐aged and older US Hispanic/Latinos

**DOI:** 10.1002/alz.71383

**Published:** 2026-04-25

**Authors:** Paola Filigrana, Melissa Lamar, Monica Batalha, Jee‐Young Moon, Wassim Tarraf, Richard B. Lipton, Linda C. Gallo, Martha L. Daviglus, Krista M. Perreira, Ariana M. Stickel, Fernando D. Testai, Bharat Thyagarajan, Hector M Gonzalez, Carmen R. Isasi

**Affiliations:** ^1^ Department of Epidemiology and Population Health Albert Einstein College of Medicine Bronx New York USA; ^2^ Rush Alzheimer's Disease Center and the Department of Psychiatry and Behavioral Sciences Rush University Chicago Illinois USA; ^3^ Institute for Minority Health Research, College of Medicine University of Illinois at Chicago Chicago Illinois USA; ^4^ Department of Healthcare Sciences, Institute of Gerontology Wayne State University Detroit Michigan USA; ^5^ Department of Neurology Albert Einstein College of Medicine Bronx New York USA; ^6^ Department of Psychology San Diego State University San Diego California USA; ^7^ Department of Social Medicine University of North Carolina at Chapel Hill Chapel Hill North Carolina USA; ^8^ Department of Neurology and Rehabilitation, College of Medicine University of Illinois at Chicago Chicago Illinois USA; ^9^ Department of Laboratory Medicine and Pathology University of Minnesota Minneapolis Minnesota USA; ^10^ Department of Neurosciences University of California San Diego California USA

**Keywords:** adults, cognitive change, cognitive function, Hispanics, Latino, life course, mild cognitive impairment, socioeconomic mobility, socioeconomic position

## Abstract

**INTRODUCTION:**

The role of life‐course socioeconomic position (SEP) in cognitive aging remains unclear. We assessed the association between life‐course SEP with cognitive change and prevalent mild cognitive impairment (MCI).

**METHODS:**

We used data from the Hispanic Community Health Study/Study of Latinos (2008 to 2011) and its Study of Latinos‐Investigation of Neurocognitive Aging (SOL‐INCA) ancillary study (2015 to 2018, *n* = 6351; aged ≥45 years). Weighted linear and logistic regression analyses estimated the association between childhood and adult SEP (low and high) and socioeconomic mobility (enduring high or low SEP, upward or downward mobility) with change in cognitive function and prevalent MCI, respectively.

**RESULTS:**

Childhood SEP was associated with greater adverse cognitive change but was not associated with prevalent MCI. Low adult SEP, downward mobility, and enduring low SEP were associated with greater adverse cognitive change. Enduring low SEP was additionally associated with higher odds of MCI.

**DISCUSSION:**

Life‐course SEP is associated with changes in cognitive aging in middle‐aged and older Hispanic/Latino adults.

## Highlights

- Life‐course SEP is associated with changes in cognitive aging in later life.
- Low childhood SEP predicts domain‐specific cognitive change but not prevalent MCI.
- Adult SEP is a stronger predictor of cognitive change than early‐life SEP.
- Downward socioeconomic mobility is associated with adverse cognitive change.
- Enduring low life‐course SEP was associated with cognitive change and prevalent MCI.

## 1 BACKGROUND

Persons of Hispanic/Latino heritage in the United States are disproportionately burdened with Alzheimer's disease and related dementias (ADRD)^1^, ^2^, ^3^ as well as associated ADRD risk factors^4^, ^5^ relative to non‐Hispanic White individuals. Furthermore, Hispanic/Latino adults will have the largest projected increase in ADRD prevalence in the next 40 years,^3^ representing a major public health problem, with important implications for the US healthcare system. Thus, the identification of drivers at different life‐course stages of these ADRD disparities is pivotal to determining targets for interventions at earlier stages of the lifespan.

The life‐course approach suggests that the burden of ADRD in later life results from exposure to risk factors across the lifespan,^6^, ^7^ broadening the focus of ADRD risk from current to lifelong exposures.^8^, ^9^ A growing body of scientific research has shown cross‐sectional associations between a disadvantaged socioeconomic position (SEP, i.e., relative lack or reduced access to social, economic, and material resources)^10^ at select life‐course stages and lower global and domain‐specific cognitive function in later life.^8^, ^9^, ^11^, ^12^, ^13^, ^14^, ^15^, ^16^, ^17^, ^18^, ^19^ However, the role of a disadvantaged life‐course SEP more broadly in cognitive decline over time and mild cognitive impairment (MCI) in later life remains unclear.^17^, ^20^, ^21^, ^22^, ^23^

Longitudinal studies across different populations have not found consistent evidence of associations between a disadvantaged life‐course SEP and cognitive decline. Some studies have shown associations between low childhood, young adulthood, or late‐life SEP and cognitive decline in later life.^9^, ^17^, ^24^ Conversely, most studies have reported no associations of low SEP at these life‐course stages and global or domain‐specific cognitive decline.^8^, ^20^, ^25^, ^26^, ^27^ Additionally, some studies have reported results that run counter to hypothesized, with faster cognitive decline associated with higher childhood^28^ and adult SEP.^20^, ^29^

Few studies have examined associations between life‐course SEP and later life MCI, which is marked by greater‐than‐expected cognitive decline without significant impairment in daily functioning. Existing studies have mostly assessed the association of adult SEP or socioeconomic mobility with prevalent or incident MCI; few studies have examined the impact of childhood SEP.^30^, ^31^, ^32^ Although these studies have shown some evidence of associations between lower childhood SEP,^32^ early to midlife SEP,^33^, ^34^ and downward socioeconomic mobility^18^, ^35^, ^36^, ^37^ with increased prevalent or incident MCI, little is known about their role in cognitive impairment among Hispanic/Latino individuals. This is particularly important, as identifying risk factors for MCI offers opportunities for earlier interventions.

In the United States, the Hispanic/Latino population experiences extensive socioeconomic disadvantage over the life course; however, most studies examining the association between life‐course SEP and cognitive change and MCI are focused on non‐Hispanic populations, with fewer studies including people of Hispanic/Latino background.^18^, ^38^ Moreover, the current literature remains inconclusive as to which period over the life‐course socioeconomic disadvantage may have the greatest impact on cognitive change and MCI. Lastly, less research has examined how life‐course socioeconomic mobility relates to cognitive change and impairment. To address these research gaps, this study assessed the association between childhood and adult SEP, as well as socioeconomic mobility from childhood to adulthood, with 7‐year changes in global and domain‐specific cognitive function and prevalent MCI among middle‐aged and older Hispanic/Latino adults.

## 2 METHODS

### 2.1 Study design and population

The Hispanic Community Health Study/Study of Latinos (HCHS/SOL) is a population‐based cohort study of Hispanic/Latino adults representative of four major urban areas (Bronx, NY; Chicago, IL; Miami, FL; San Diego, CA) and several heritage groups (Central American, Cuban, Mexican, Puerto Rican, Dominican, and South American).^39^ Middle‐aged and older adults (45 to 74 years) underwent cognitive examination at HCHS/SOL baseline (2008 to 2011, *n* = 9652). As part of the SOL‐Investigation of Neurocognitive Aging (SOL‐INCA) ancillary study, a second visit included cognitive testing conducted ∼7 years later (2015 to 2018) with 6377 eligible HCHS/SOL participants.^6^ Eligibility criteria for SOL‐INCA included (1) HCHS/SOL visit 2 (2014 to 2017) completion, (2) cognitive testing at baseline, and (3) age 50 years or older. In the current study, we conducted analysis using data from 6351 participants with data from both cognitive assessments. HCHS/SOL and SOL‐INCA were reviewed and approved by each participating institution's Institutional Review Board. Written informed consent was obtained from all participants.

### 2.2 Measurements of life‐course SEP

*Childhood SEP*: Following previous work,^19^, ^40^ we used participants’ self‐report of their father's and mother's educational attainment as a proxy for childhood SEP. Specifically, we selected the highest education achieved by either the father or mother and classified parental education as high school or above and less than high school. Since maternal and paternal education may differentially influence cognitive change and MCI, we also evaluated these exposures individually.

*Adult SEP*: We conceptualized SEP in adulthood as a composite index encompassing indicators of economic, social, and work status, as well as assets.^10^, ^41^ We included participants’ self‐reported education, household income, occupation, and house ownership to represent these dimensions of SEP at HCHS/SOL baseline. Educational attainment was scored as less than high school (0), high school (1), and more than high school (2); annual household income was scored as < $30,000 (0), $30,000 to $50,000 (1), > $50,000 (2); current occupation was scored as unemployed and looking for a job (0), not in the labor force including homemaker and retired (1), non‐skilled, service, or skilled worker (2), and professional or managerial (3); and house ownership was scored as renting with no home (0) and homeowner with or without a mortgage (1). We summed across indicators to obtain the index, ranging from 0 to 8 (higher scores indicate higher adult SEP). Then we categorized the index as high SEP (index 3 to 8) and low SEP (index < 3) based on the median.

*Socioeconomic mobility*: To ascertain socioeconomic mobility from childhood to adulthood, we combined childhood (parental education less than high school or high school or above) and adult SEP (low or high SEP) at HCHS/SOL baseline and classified participants into four socioeconomic mobility categories: enduring low SEP (low childhood and low adult SEP), upward mobility (low childhood to high adult SEP), downward mobility (high childhood to low adult SEP), and enduring high SEP (high childhood and high adult SEP).^19^, ^40^

**RESEARCH IN CONTEXT**

1. **Systematic review**: The authors conducted a literature review using PubMed. Evidence on the associations between childhood SEP and cognitive change or MCI remains inconclusive. In contrast, low adult SEP seems to be a strong predictor of cognitive decline in later life. Socioeconomic mobility has been associated with both cognitive decline and MCI.
2. **Interpretation**: Our findings support the idea that low SEP over the life course is associated with adverse changes in global and specific domains of cognitive function in middle‐aged and older Hispanic/Latino adults. Less consistent evidence was found for prevalent MCI.
3. **Future directions**: Further longitudinal studies are needed to shed light on the association between early‐life SEP and trajectories of cognitive decline. Studies are also needed to assess the role of life‐course SEP in incident MCI and transitions to dementia. Further research should investigate how resilience factors, such as social support, mitigate the impact of socioeconomic disadvantages on cognitive aging.

### 2.3 Measures of cognitive function and MCI

*Cognitive function*: Five standardized cognitive tests representing different domains of cognitive function were administered at HCHS/SOL baseline and the SOL‐INCA follow‐up visit.^6^, ^42^ These five tests included the two scores from the Brief‐Spanish English Verbal Learning Test (B‐SEVLT), a measure of verbal learning and memory, specifically, the B‐SEVLT sum across the three learning trials and the B‐SEVLT post‐interference recall score^43^; the Controlled Oral Word Association or Word Fluency Test (WF),^44^ total words for letters “F” and “A” the Digit Symbol Substitution (DSS) Test of the Wechsler Adult Intelligence Scale‐Revised, a measure of psychomotor speed^45^; and a Six Item Screener (SIS), a brief measure of mental status.^46^ These tests were administered in both visits by trained technicians in Spanish or English based on the participants' preference – although code switching for WF was allowed. Each neurocognitive test was z‐score standardized for both visits using the baseline mean and standard deviation (SD). We averaged across the four domain‐specific cognitive tests (excluding the SIS) to obtain a measure of global cognition for each visit. Change in global cognition and domain‐specific cognitive function was calculated as the difference between the follow‐up visit and baseline.

*Prevalent MCI*: Prevalent cases of MCI were identified at the SOL‐INCA follow‐up visit.^6^ The battery used included the aforementioned tests (i.e., B‐SEVLT, WF, and DSS) obtained at both visits, as well as the extended SOL‐INCA battery, for example, the Trail Making Test (TMT, Parts A and B), the National Institutes of Health Toolbox Picture Vocabulary Test (PVT), measures of self‐reported cognitive decline (i.e., Everyday Cognition‐12; ECog12), and instrumental activities of daily living (IADLs).^47^, ^48^ Using the baseline cognitive function battery and the SOL‐INCA battery, participants were classified as having prevalent MCI according to the following four National Institute on Aging‐Alzheimer's Association core criteria^49^: (1) any cognitive test score in the mildly impaired range (i.e., lower than or equal to −1 SDs) relative to the SOL‐INCA robust internal norms accounting for age‐, education‐, sex‐, and PVT‐adjusted scores; (2) significant cognitive decline from baseline (i.e., at least −0.055 SD/year); (3) self‐reported cognitive decline (based on ECog12); and (4) no or minimum IADL impairment.^6^, ^42^, ^50^

### 2.4 Covariates

Based on previous studies, we included demographic, behavioral, and clinical variables collected at baseline as covariates, which may be related to each measure of life‐course SEP and are known risk factors for cognitive function and MCI (Figure S1).^7^, ^51^, ^52^ Specifically, for childhood SEP measures, we accounted for year of birth (linear), sex (male and female), Hispanic/Latino background (Dominican, Cuban, Mexican, Puerto Rican, Central American, South American, and mixed or other), and place of birth (born in the 50 US states/DC and born outside the 50 US states/DC). For adult SEP, we additionally accounted for childhood SEP (parental education less than high school and high school or above), age of immigration (born in the 50 US states/DC, born outside the 50 US states/DC and age of immigration <12 years, and born outside the 50 US states/DC and age of immigration ≥12 years), field center (Chicago, Bronx, San Diego, and Miami), language preference (English and Spanish), marital status (single, married, or living with a partner, and separated or widow/widower), health insurance coverage (yes and no), smoking status (never/former and current), alcohol use level (no current use, low‐risk drinker, and at‐risk drinker based on sex‐specific cutoff for weekly alcohol use from the National Institute on Alcohol Abuse and Alcoholism),^53^ physical activity level (measured through accelerometer for 7 days and classify as inactive or low activity [no activity beyond baseline activities of daily living^54^ or activity beyond baseline but <150 min of moderate‐intensity activity a week], medium activity [150 to 300 min of moderate activity a week or 75 to 150 min of vigorous activity a week], and high activity [> 300 min of moderate activity a week or >150 min of vigorous activity a week or an equivalent combination of both]); ^55^ depression symptoms based on the 10‐item Center for Epidemiological Studies Depression Scale (CESD‐10),^56^ hypertension (yes and no based on measured systolic and diastolic blood pressure ≥140/90 mmHg or blood pressure‐lowering medication use), diabetes (no diabetes, pre‐diabetes, and diabetes based on the definition of the American Diabetes Association or glucose‐lowering medication use); ^57^ prevalent CVD or stroke (yes and no based on a combination of identified possible old myocardial infarction on baseline electrocardiogram, or self‐report of medical diagnosis of a heart attack, cardiovascular procedures, or self‐report of medical diagnosis of stroke), and self‐reported health status (excellent/good, fair, and poor based on self‐reported health as part of the Short‐Form 12‐Item [version 2] Health Survey [SF‐12 v2]). A similar rationale was followed to select the covariates for socioeconomic mobility.

### 2.5 Statistical analysis

We described the distribution of demographic, behavioral, and health characteristics weighted to the target population of SOL‐INCA. To quantify the association between each measure of life‐course SEP and 7‐year change in global cognition and specific cognitive domains, as well as prevalent MCI, we fitted weighted regression models, accounting for the factors of the complex sampling design (i.e., clustering, stratification, and probability sampling weights). Given the life‐course approach where covariates downstream of childhood SEP (e.g., adult SEP, age of immigration, clinical factors) may act as mediators, their direct inclusion in the regression models may result in biased estimates (Figure S1).^58^, ^59^, ^60^ To address this, following the approach by Robins et al.,^58^ we conducted inverse probability weighting (IPW) and marginal structural models to estimate controlled direct effects of each measure of life‐course SEP on cognitive change and prevalent MCI. First, we calculated stabilized IPW (SIPW) for each life‐course SEP measure using logistic regression models. The numerator of the SIPW was estimated as the predicted probability of exposure (low vs high SEP) given SEP at the previous life‐course stage and relevant baseline confounders, including year of birth, sex, place of birth, and Hispanic/Latino background. The denominator of the SIPW was estimated as the predicted probability of exposure given SEP at the previous life‐course stage and the specific set of confounders for each SEP measure. The SIPW for each life‐course SEP measure was then multiplied by the sampling weights to obtain total SIPWs. Since the sampling weights in SOL‐INCA accounted for differential loss to follow‐up, their inclusion accounted for selective attrition. Then, using the calculated SIPW along with the factors of the complex sampling design (i.e., clustering, stratification, and sampling probability weights), we fitted weighted linear and logistic regression models to estimate the association between each life‐course SEP exposure with change in measures of cognitive function and prevalent MCI, respectively, represented as beta coefficients or odds ratios (ORs) and their 95% confidence intervals (CIs). In the regression models for change in cognitive function, we adjusted in addition for elapsed time between baseline and follow‐up visit and the cognitive function measure at baseline. As recommended by Robins et al.,^58^ in all regression models, we additionally adjusted for the relevant confounders included in the numerator of the SIPW.^61^ In our regression models, Model 1 shows the estimated total effect of each measure of childhood SEP on cognitive change or prevalent MCI (i.e., direct childhood SEP effect and indirect effect through adult SEP). Model 2 shows the estimated controlled direct effects of childhood and adult SEP on cognitive change or prevalent MCI (i.e., direct childhood SEP effect not through adult SEP). We adjusted the *p* values for false discovery rate (FDR) using the Benjamini–Hochberg correction method.^62^

Additionally, as in this population, age, sex, or immigration characteristics may play a role in the association between life‐course SEP and cognitive change and prevalent MCI, we evaluated the interaction of childhood or adult SEP or socioeconomic mobility with age, sex, place of birth, and age of immigration by adding interaction terms individually in our weighted regression models.

Since there were missing data in parental educational attainment (13%), annual household income (8%), and physical activity (15%), we conducted multiple imputation, using sequential imputation and chained equations from a set number of trials to obtain 50 sets of complete imputed data sets.^63^, ^64^ All analyses were conducted using these imputed datasets and summary estimates and 95% CIs for the associations were obtained accounting for the within‐ and between‐imputation variability. All analyses were conducted using Stata (StataCorp, LLC, College Station, TX, USA).^65^

In sensitivity analysis, we used an alternative definition of socioeconomic mobility based on consistent proxies for childhood and adult SEP. In this analysis, we combined parental educational attainment as a proxy for childhood SEP and participants’ educational attainment as a proxy for adult SEP (classified as less than high school and high school or above). Then we classified participants into the four categories of socioeconomic mobility. In addition, since education is a strong predictor of cognition, we built an alternative index of adult SEP excluding participant's educational attainment. Then participant education was included as a predictor in the IPW estimation for adult SEP.

## 3 RESULTS

Characteristics at HCHS/SOL baseline are shown in Table 1. The mean age at baseline was 56 years (SD ± 8.1), about half were female (54.5%), 32.8% were of Mexican background, and most were foreign‐born with immigration to the United States at 12 years of age or older. Most of the population (64.4%) had parents with less than a high school education, and more than a third (39.7%) had low adult SEP. Regarding socioeconomic mobility, 35.3% experienced upward socioeconomic mobility and 28.3% remained in a low SEP over their life course (Table 1).

**TABLE 1:** Sociodemographic, behavioral, and clinical characteristics of Hispanic/Latino adults, study of Latinos‐Investigation of Neurocognitive Aging (unweighted *n* = 6351).

Characteristics	Unweighted, *n*	Weighted percentage or weighted mean (SD)
**Demographic characteristics**		
Age, mean (SD)		56.4 (8.1)
Sex, No. (%)		
Female	4092	54.5
Male	2259	45.5
Hispanic/Latino background, No. (%)		
Dominican	573	9.5
Central American	630	7.3
Cuban	1054	25.9
Mexican	2438	32.8
Puerto Rican	1061	15.4
South American	461	5.2
Mixed/Other	120	3.9
Field center, No. (%)		
Bronx	1418	27.3
Chicago	1580	12.5
Miami	1706	36.3
San Diego	1647	23.8
Age of immigration to the 50 US states/DC, No. (%)		
Born in 50 US States/DC	556	8.3
Born outside 50 US States/DC and immigration <12 years of age	375	5.9
Born outside 50 US States/DC and immigration ≥12 years of age	5400	85.8
Language preference, No. (%)		
Spanish	5556	87.4
English	795	12.6
Marital status, No. (%)		
Single	1064	16.9
Married or living with a partner	3499	53.9
Separated, divorced, or widow(er)	1772	29.1
Health insurance, No. (%)		
Yes	3452	57.2
No	2842	42.7
**Behavioral and clinical characteristics**		
Physical activity level, No. (%)		
Inactive or low	3600	65.1
Moderate	1037	19.9
High	735	14.9
Smoking status, No. (%)		
Never or former	5233	81.5
Current	1107	18.5
Alcohol use level, No. (%)		
No current use	3614	54.6
Low‐risk drinker	2490	41.6
At‐risk drinker	237	3.8
Depression symptoms (CESD‐10), mean (SD)		7.4 (6.3)
Diabetes, No. (%)		
No diabetes	1685	24.6
Prediabetes	2901	46.5
Diabetes	1763	28.9
Hypertension, No. (%)	2777	47.1
Prevalent CVD, No. (%)	503	9.1
Self‐rated health, No. (%)		
Excellent or good	4077	64.3
Fair	1853	28.9
Poor	405	6.8
Life‐course SEP measures		
Childhood SEP, No. (%)		
Parental education		
High school or above	1759	35.6
Less than high school	3895	64.4
Maternal education		
High school or above	1207	25.0
Less than high school	4327	74.9
Paternal education		
High school or above	1387	31.7
Less than high school	3685	68.3
Adult SEP, No (%)		
High SEP	3486	60.3
Low SEP	2310	39.7
Socioeconomic mobility, No. (%)		
Enduring high SEP	1224	27.7
Upward mobility	2046	35.3
Downward mobility	425	8.6
Enduring low SEP	1533	28.3

Abbreviations: CESD‐10, 10‐item Center for Epidemiological Studies Depression Scale; CVD, cardiovascular disease; *n*, sample size; SD, standard deviation; SEP, socioeconomic position.

The total estimated effect of each measure of childhood SEP (i.e., direct childhood SEP effect and indirect effect through adult SEP) on cognitive change and prevalent MCI are shown in Model 1 of Table 2. In contrast, Model 2 shows the estimated controlled direct effect of each measure of childhood or adult SEP (i.e., direct childhood SEP effect not through adult SEP) on cognitive change and prevalent MCI. We did not find evidence of an association between parental education and 7‐year changes in global cognition, verbal learning, verbal memory, or prevalent MCI. However, adults whose parents had less than high school education had a greater adverse change in specific cognitive domains, including word fluency (*β* for WF: −0.07; 95% CI: −0.13, −0.00), processing speed (*β* for DSS: −0.08; 95% CI: −0.13, −0.02), and mental status (*β* for SIS: −0.12; 95% CI: −0.20, −0.03) relative to those whose parents had high school or above. These associations appear to be primarily explained by maternal education, as we found a more pronounced adverse change in global cognition and specific cognitive domains among adults whose mothers had lower education levels compared to those whose mothers had higher education levels (Model 1). Additionally, adult SEP seems to partially mediate these associations (Model 2). Most of these associations remain after FDR adjustment, except for parental or paternal education and word fluency (Table S1).

**TABLE 2:** Association between childhood and adult socioeconomic position with change in cognitive function and prevalent mild cognitive impairment, Study of Latinos‐Investigation of Neurocognitive Aging.

Outcome	Childhood SEP			Adult SEP
	Parental education	Maternal education	Paternal education	
**Change in cognitive function, *β* (95% CI)**				
∆Global cognition				
Model 1^*^	−0.03 (−0.07, 0.02)	−0.08 (−0.13, −0.03) ^┼^	−0.01 (−0.07, 0.04)	
Model 2^*^	−0.02 (−0.06, 0.03)	−0.06 (−0.12, −0.01) ^┼^	−0.00 (−0.05, 0.05)	−0.10 (−0.14, −0.05)‡
∆B‐SEVLT sum				
Model 1^*^	−0.02 (−0.09, 0.05)	−0.03 (−0.14, 0.07)	−0.01 (−0.10, 0.09)	
Model 2^*^	0.01 (−0.07, 0.08)	0.00 (−0.10, 0.11)	0.03 (−0.06, 0.12)	−0.16 (−0.23, −0.09)‡
∆B‐SEVLT recall				
Model 1^*^	−0.04 (−0.11, 0.03)	−0.11 (−0.21, −0.01) ┼	−0.03 (−0.12, 0.05)	
Model 2^*^	−0.02 (−0.09, 0.05)	−0.09 (−0.18, 0.01)	−0.00 (−0.09, 0.08)	−0.11 (−0.18, −0.04)^┼^
∆WF				
Model 1^*^	−0.07 (−0.13, −0.00)^┼^	−0.19 (−0.28, −0.09) ‡	−0.10 (−0.19, −0.02) ^┼^	
Model 2^*^	−0.05 (−0.11, 0.02)	−0.16 (−0.25, −0.07) ‡	−0.08 (−0.16, 0.01)	−0.14 (−0.20, −0.08)‡
∆DSS				
Model 1^*^	−0.08 (−0.13, −0.02)^┼^	−0.10 (−0.15, −0.04) ^┼^	−0.07 (−0.12, −0.01) ^┼^	
Model 2^*^	−0.07 (−0.12, −0.01)^┼^	−0.09 (−0.14, −0.03)^┼^	−0.06 (−0.11, −0.00)	−0.11 (−0.15, −0.06)‡
∆SIS				
Model 1^*^	−0.12 (−0.20, −0.03)^┼^	−0.08 (−0.18, 0.02)	−0.08 (−0.15, 0.0)	
Model 2^*^	−0.10 (−0.19, −0.01)^┼^	−0.06 (−0.16, 0.05)	−0.05 (−0.14, 0.05)	−0.09 (−0.18, 0.00)
Prevalent MCI, OR (95% CI)				
Model 1^*^	1.21 (0.92, 1.59)	1.27 (0.90, 1.78)	1.21 (0.89, 1.63)	
Model 2^*^	1.15 (0.85, 1.54)	1.20 (0.84, 1.71)	1.15 (0.84, 1.57)	1.27 (0.99, 1.64)

Abbreviations: B‐SEVLT, Brief‐Spanish English Verbal Learning Test; CI, confidence interval; DSS, Digit Symbol Substitution; MCI, mild cognitive impairment; OR, odds ratio; SEP, socioeconomic position; SIS, six‐item screener; WF, word fluency.

* The outcome regression models were weighted with the stabilized inverse probability weights to adjust for the set of confounders defined for each measure of life‐course SEP. We also included directly in the outcome regression models the relevant baseline confounders (year of birth, sex, place of birth, and Hispanic/Latino background) used to estimate the numerator of the inverse probability weights. Model 1 shows the estimated total effect of each measure of childhood SEP (i.e., direct childhood SEP effect and indirect effect through adult SEP) on cognitive change and prevalent MCI. Model 2 shows the estimated controlled direct effects of each measure of childhood and adult SEP (i.e., direct childhood SEP effect not through adult SEP) on cognitive change and prevalent MCI. Childhood SEP: parental education, maternal education, and paternal education (reference: high school or above), adult SEP (reference: high SEP). Prevalent MCI: proportion of total MCI cases identified at SOL‐INCA ancillary study visit).

^┼^ *P* value: 0.05 to 0.001

^‡^ *P* < 0.001.

Regardless of SEP in childhood, adult SEP was associated with 7‐year change in cognitive function but was not associated with prevalent MCI (Model 2). Specifically, individuals with low SEP in adulthood had greater adverse change in global cognition (*β*: −0.10, 95% CI: −0.14, −0.05) and specific domains of cognitive function, including verbal learning (*β* for B‐SEVLT sum: −0.16; 95% CI: −0.23, −0.09), verbal memory (*β* for B‐SEVLT recall: −0.11; 95% CI: −0.18, −0.04), word fluency (*β* for WF: −0.14; 95% CI: −0.20, −0.08), and processing speed (*β* for DSS: −0.11; 95% CI: −0.15, −0.06) relative to individuals with high adult SEP. These associations remained significant after adjusting for FDR (Table S1). We also found consistent results, although attenuated, in sensitivity analysis that removed participant education from the index of adult SEP and accounted for education as a covariate (Table S2).

We found similar associations between socioeconomic mobility and 7‐year cognitive change (Table 3). Hispanic/Latino adults with enduring low SEP from childhood to adulthood relative to adults with enduring high SEP showed a greater adverse change in global cognition (*β*: −0.11; 95% CI: −0.17, −0.04) and all domains of cognitive function: verbal learning (*β* for B‐SEVLT sum: −0.14; 95% CI: −0.24, −0.04), verbal memory (*β* for B‐SEVLT recall: −0.12; 95% CI: −0.21, −0.03), word fluency (*β* for WF: −0.18; 95% CI: −0.27, −0.09), processing speed (*β* for DSS: −0.18; 95% CI: −0.25, −0.11), and mental status (*β* for SIS: −0.17; 95% CI: −0.27, −0.06). The population with enduring low SEP also had greater odds of prevalent MCI. Similarly, adults with downward socioeconomic mobility also had a greater adverse change in global cognition and cognitive‐specific domains, except for verbal memory and mental status compared to adults with enduring high SEP. These associations remained significant after adjusting for FDR (Table S1). We found consistent results, except for MCI, in a sensitivity analysis that used socioeconomic mobility based on parental and participant education as proxies for childhood and adult SEP, respectively (Table S3).

**TABLE 3:** Association between socioeconomic mobility with change in cognitive function and prevalent mild cognitive impairment, Study of Latinos‐Investigation of Neurocognitive Aging.

Cognitive function	Upward mobility^*^	Downward mobility^*^	Enduring low SEP ^*^
**Change in cognitive function, *β* (95% CI)**			
∆Global cognition	−0.01 (−0.07, 0.05)	−0.13 (−0.23, −0.03) ┼	−0.11 (−0.17, −0.04)^┼^
∆B‐SEVLT sum	0.02 (−0.07, 0.12)	−0.19 (−0.32, −0.05)^┼^	−0.14 (−0.24, −0.04)^┼^
∆B‐SEVLT recall	0.00 (−0.09, 0.09)	−0.10 (−0.25, 0.04)	−0.12 (−0.21, −0.03)^┼^
∆WF	−0.03 (−0.11, 0.05)	−0.17 (−0.31, −0.03)^┼^	−0.18 (−0.27, −0.09)‡
∆DSS	−0.08 (−0.14, −0.02)^┼^	−0.17 (−0.27, −0.07)^┼^	−0.18 (−0.25, −0.11)‡
∆SIS	−0.05 (−0.15, 0.05)	−0.01 (−0.16, 0.13)	−0.17 (−0.27, −0.06)^┼^
Prevalent MCI, OR (95% CI)	1.18 (0.78, 1.78)	1.64 (0.93, 2.91)	1.47 (1.02, 2.12)^┼^

Abbreviations: B‐SEVLT, Brief‐Spanish English Verbal Learning Test; CI, confidence interval; DSS, Digit Symbol Substitution; MCI, mild cognitive impairment; SIS, six‐item screener; WF, Word fluency.

* The outcome regression models were weighted with the stabilized inverse probability weights to adjust for the set of covariates defined for socioeconomic mobility. We also included directly in the outcome regression models the baseline confounders included to estimate the numerator of the inverse probability weights for socioeconomic mobility (year of birth, sex, place of birth, and Hispanic/Latino background). Socioeconomic mobility (reference: Enduring high SEP: high childhood and high adult SEP), upward mobility: low childhood to high adult SEP, downward mobility: high childhood to low adult SEP, enduring low SEP: low childhood and low adult SEP. Prevalent MCI: proportion of total MCI cases identified at SOL‐INCA ancillary study visit).

^┼^ *P* = 0.05 to 0.001

^‡^ *P* < 0.001.

We did not find evidence of interaction between childhood or adult SEP or socioeconomic mobility and age, sex, place of birth, and age of immigration to the United States (data not shown).

## 4 DISCUSSION

In this population‐based cohort study of middle‐aged and older Hispanic/Latino adults, we found that life‐course SEP was associated with 7‐year change in global cognition and specific domains of cognitive function in later life. Specifically, we found that low early‐life SEP was associated with greater adverse change in global cognition, verbal memory, word fluency, psychomotor speed, and mental status scores, but it was not associated with changes in verbal learning. These associations appeared to be primarily explained by maternal education. We also found that low SEP in adulthood was associated with greater adverse change in global cognition, verbal learning and memory, word fluency, and psychomotor speed. Our results also show that downward socioeconomic mobility and enduring low SEP from childhood to adulthood were associated with greater 7‐year adverse global and domain‐specific cognitive change. Enduring low SEP was additionally associated with higher odds of prevalent MCI. Overall, results from this study support the idea that a disadvantaged SEP over a person's lifespan is associated with cognitive aging in later life.

The lack of identified association of early‐life SEP with changes in verbal learning contrasts with findings from some prior studies^8^, ^24^, ^25^, ^26^, ^27^, ^29^, ^66^, ^67^, ^68^ but not others.^9^, ^17^, ^20^, ^69^, ^70^ Likewise, our results linking early‐life socioeconomic disadvantage with greater adverse change in mental status^69^, ^71^ and word fluency^69^ are in line with results from Tsang et al.^69^ and Maurice et al.^71^ but differ from other studies.^9^, ^25^, ^29^, ^70^, ^72^ The association between childhood SEP and later‐life prevalent MCI is rarely examined.^30^, ^31^, ^32^ Unlike our findings, Zhang et al.^32^ found that low childhood SEP was significantly associated with a higher risk of later‐life incident MCI. However, their population was largely from a rural setting (78%), with lower educational attainment (13% reported high school or above), and worse self‐reported health status (73% reported poor health), which may explain discrepancies in findings. Additionally, their study included MCI measurements across several follow‐up waves, and it is possible that associations of childhood SEP with incident MCI will be observed in the HCHS/SOL population with greater duration of follow‐up. More generally, discrepancies in findings across studies could reflect a number of influences, including differences in study designs, populations (i.e., age, sex, and race and ethnicity distributions, rural vs urban populations), and indicators of childhood SEP (i.e., parental education and occupation, economic hardship, poverty, family income, food insecurity; the use of single vs composite SEP measures). Additionally, there is variability across studies in the cognitive tests administered, domains of cognitive function evaluated, and/or criteria used to ascertain prevalent versus incident MCI. Finally, there are study differences in the timing of exposure and outcome ascertainment. Further harmonized studies are needed to shed light on these associations.

Results from this study linking lower adult SEP with greater adverse change in global cognition and specific domains of cognitive function are, however, in line with most previous studies.^17^, ^20^, ^26^, ^29^, ^73^ These associations have been observed across studies that vary in timing of adult SEP ascertainment (i.e., young‐adulthood, midlife, and late‐life), use of single indicators (e.g., educational attainment, family income, wealth)^17^, ^20^, ^26^ versus composite SEP measures,^29^, ^73^ and specific cognitive domains evaluated (e.g., global cognition, verbal memory, and processing speed). A singular study using similar statistical methods for causal inference did not find associations between adult SEP – specifically, young‐adult (i.e., years of education), midlife (i.e., family income), or late‐life (i.e., family income) SEP – and decline in global cognition over a median of 6‐year follow‐up.^8^ However, their study population was mostly non‐Hispanic White (92%), highly educated (14.4 ± 3 years), and older (mean age 80 ± 6 years at baseline)^74^ – factors that may have reduced the variance in cognitive decline experienced.^8^ Studies examining the association between adult SEP and MCI are scarce,^31^, ^33^, ^75^ with most combining MCI and dementia as the outcome of interest.^31^, ^34^ The lack of association found in the current study is consistent with some previous research^33^, ^75^ but differs from others.^31^, ^34^ Our younger study population (mean age: 56 ± 8 years), compared with previous research primarily focused on adults 65 years and older, may have limited the identification of MCI cases and contributed to the lack of association observed in this study.

Our results showing that socioeconomic mobility from childhood to adulthood was associated with both change in cognitive function and MCI are consistent with most previous studies.^17^, ^18^, ^20^, ^35^, ^36^, ^37^ Across studies, individuals with enduring low SEP or downward socioeconomic mobility over the life course, relative to those with enduring high SEP, experienced steeper declines in global cognition or specific cognitive function domains^17^, ^20^ and higher risk of prevalent or incident MCI.^18^, ^35^, ^36^, ^37^, ^76^ In contrast, no consistent evidence has been found for an association of life‐course upward socioeconomic mobility with changes in cognitive function or MCI.^18^, ^20^, ^37^, ^76^

Collectively, our findings support a life‐course perspective to elucidate the impact of SEP across the lifespan on cognitive aging and risk for dementia in later life. We found evidence that a disadvantaged SEP in early life, primarily indicated by low maternal education, has a long‐lasting influence on adverse cognitive change with indirect pathways through SEP in adulthood. This suggests that growing up in a socioeconomically disadvantaged environment, particularly with less educated mothers, who are often the primary caregivers, may reflect a less cognitively stimulating home environment, less nutritious diet or food insecurity, and limited access to material resources.^24^, ^77^ These factors may negatively impact brain development in early life, which may contribute to adverse cognitive aging in later life.^24^, ^28^, ^66^, ^77^, ^78^, ^79^ Moreover, given the strong and independent association between SEP in adulthood and global cognition, as well as most cognitive function domains, adult SEP appears to be a robust predictor of cognitive change in later life. This suggests that a disadvantageous SEP in adulthood, as reflected in low educational attainment and occupational achievement, as well as limited access to material resources, may lead to adverse cognitive changes over time.^17^, ^20^, ^23^ Additionally, our findings support that adult SEP might be one pathway through which childhood SEP impacts cognitive change in later life. More importantly, our results showing greater cognitive aging among individuals enduring socioeconomic disadvantage throughout the life course (i.e., enduring low SEP) suggest that experiences of low SEP at several life stages may build up greater disadvantages over time, contributing to greater cognitive deterioration in later life.^8^, ^28^ Furthermore, the adverse cognitive changes associated with downward socioeconomic mobility suggest that socioeconomic disadvantages in adulthood may dilute the benefits provided by an advantageous SEP in early life. In addition, our findings showing no differences in cognitive change between those with an upward socioeconomic mobility, compared to those with enduring high SEP, suggest that moving up to a more advantageous SEP in adulthood from a low childhood socioeconomic background may improve access to better living conditions and social and economic resources, which, in turn, may positively influence cognitive aging. These findings are consistent with the hypothesis that adult SEP is a robust predictor of cognitive aging in later life.

This study has some limitations. Given the lack of other relevant measures (e.g., parental occupation, family income, assets), we used participants’ self‐reports of their father’ and/or mother's educational attainment as a proxy for childhood SEP, which may fail to capture the complexity of a socioeconomically disadvantaged environment in early life. Moreover, this information was collected retrospectively at baseline in all participants regardless of cognitive status, which may be subject to recall bias that, in turn, may influence our estimated associations. Additionally, our follow‐up time was relatively short (7 years), which might have limited our ability to evaluate the influence of life‐course SEP on trajectories of cognitive change over time. Similarly, the focus on prevalent MCI and the absence of longitudinal data on MCI hinder our ability to assess the association between life‐course SEP and MCI risk. Furthermore, given the relatively young population with few prevalent MCI cases, our analysis might be underpowered to find significant associations with this definition of MCI. Finally, although our analyses included widely recognized confounders of the main associations, results from our marginal structural models and IPW depend on the assumption of no unmeasured confounding, which cannot be assured, as we did not account for health status during childhood or abuse, neglect, or household dysfunction in early life.

Despite these limitations, this study has several strengths. This study provides evidence on the role of life‐course SEP on cognitive change and prevalent MCI in a large, well‐characterized population of community‐based US Hispanic/Latino adults. The use of marginal structural models and IPW helped to estimate direct and indirect pathways across measures of life‐course SEP, while controlling for confounding, which reduces bias compared to conventional regression approaches. This approach also helped to disentangle the contribution of childhood from adult SEP. Our findings were robust to alternative measures of adult SEP and socioeconomic mobility since consistent results were found when participant's education was removed from the index of adult SEP and when parents’ and participants’ education were used as proxies for childhood and adult SEP, respectively. Moreover, our definition of MCI was based on criteria from NIA‐AA,^49^ which includes a combination of objective measures of cognitive decline, functional impairment, and subjective reported cognitive decline.

In conclusion, this study found evidence that SEP throughout the life course is associated with 7‐year cognitive change and prevalent MCI in middle‐aged and older Hispanic/Latino adults. Results from this study have important public health implications, as policies that alleviate life‐course socioeconomic disadvantage ensure access to education across the life course and promote opportunities for social mobility may support healthy cognitive aging.

## CONFLICT OF INTEREST STATEMENT

The authors declare no conflicts of interest. Author disclosures are available in the Supporting Information.

## CONSENT STATEMENT

The HCHS/SOL and SOL‐INCA ancillary study were reviewed and approved by each participating institution's Institutional Review Board. Written informed consent was obtained from all participants.

## Supporting information



Supporting Information

Supporting Information
