# Supplementary material for: The role of life‐course socioeconomic position in cognitive change and mild cognitive impairment among middle‐aged and older US Hispanic/Latinos
Source: Alzheimers Dement. 2026 Apr 25;22(4):e71383. doi: 10.1002/alz.71383 (PMC13109640; doi:10.1002/alz.71383)
Supplement: Supplementary file 1 — Supporting Information [file ALZ-22-e71383-s002.docx]

**Supplementary online materials**

**Figure S1. Hypothesized causal relationships between measures of life-course SEP, confounders, and cognitive aging outcomes**

**
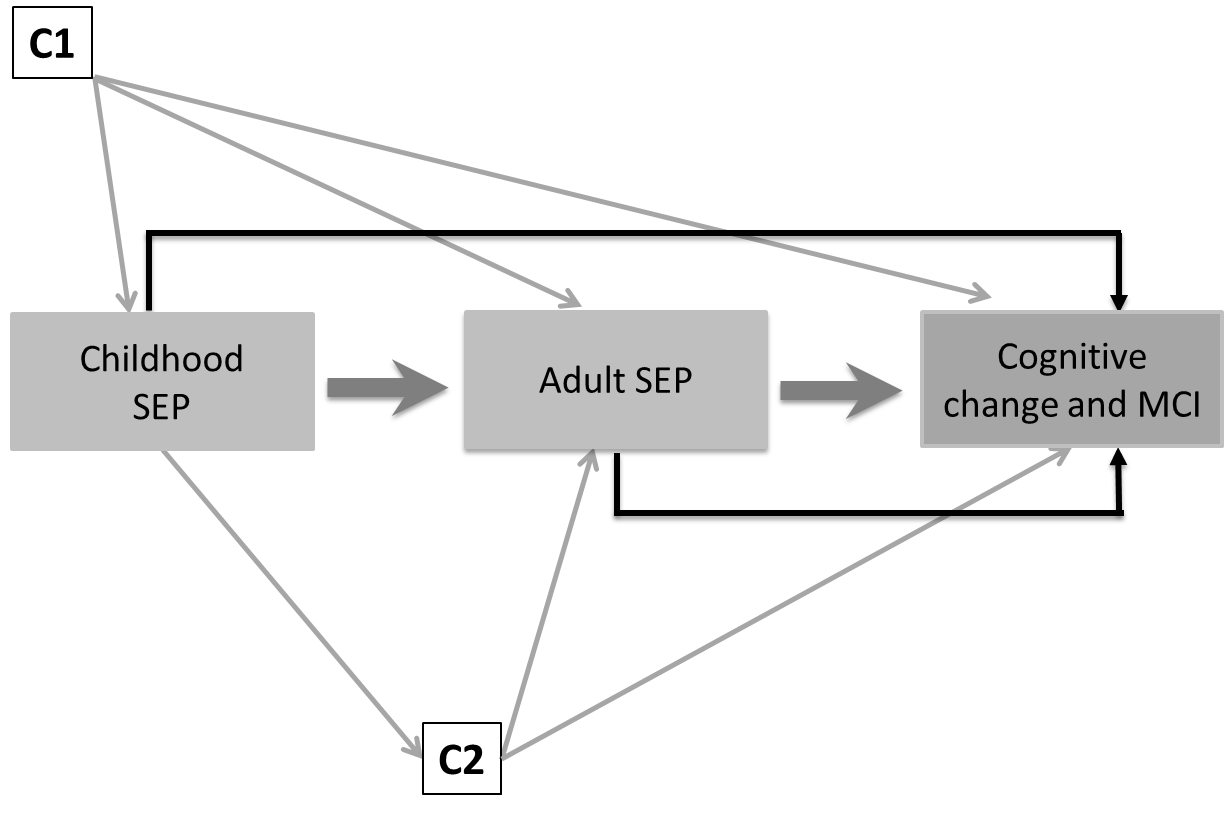
**

C1: year of birth, sex, Hispanic/Latino background, place of birth. C2: childhood SEP, age of immigration, field center, language preference, marital status, health insurance coverage, smoking status, alcohol use, physical activity, depression symptoms, hypertension, diabetes, prevalent CVD or stroke, self-reported health status

**Table S1. Adjusted p-values for false discovery rate using Benjamini-Hochberg correction method**

| **Outcome** | **Childhood SEP** | | | **Adult SEP** | **Socioeconomic mobility** | | |
| --- | --- | --- | --- | --- | --- | --- | --- |
|  | **Parental education** | **Maternal education** | **Paternal education** |  | **Upward mobility** | **Downward mobility** | **Enduring low SEP** |
| ∆Global cognition |  |  |  |  |  |  |  |
| Model 1 | 0.274 | 0.005 | 0.702 |  | 0.900 | 0.016 | 0.002 |
| Model 2 | 0.611 | 0.028 | 0.986 | 0.000 |  |  |  |
| ∆B-SEVLT sum |  |  |  |  |  |  |  |
| Model 1 | 0.602 | 0.550 | 0.857 |  | 0.898 | 0.016 | 0.008 |
| Model 2 | 0.850 | 0.937 | 0.767 | 0.000 |  |  |  |
| ∆B-SEVLT recall |  |  |  |  |  |  |  |
| Model 1 | 0.274 | 0.040 | 0.654 |  | 0.989 | 0.210 | 0.013 |
| Model 2 | 0.611 | 0.138 | 0.986 | 0.003 |  |  |  |
| ∆WF |  |  |  |  |  |  |  |
| Model 1 | 0.075 | 0.000 | 0.066 |  | 0.898 | 0.022 | 0.000 |
| Model 2 | 0.359 | 0.000 | 0.294 | 0.000 |  |  |  |
| ∆DSS |  |  |  |  |  |  |  |
| Model 1 | 0.021 | 0.003 | 0.066 |  | 0.072 | 0.006 | 0.000 |
| Model 2 | 0.077 | 0.007 | 0.294 | 0.000 |  |  |  |
| ∆SIS |  |  |  |  |  |  |  |
| Model 1 | 0.021 | 0.175 | 0.317 |  | 0.899 | 0.843 | 0.002 |
| Model 2 | 0.077 | 0.353 | 0.652 | 0.183 |  |  |  |
| Prevalent MCI |  |  |  |  |  |  |  |
| Model 1 | 0.274 | 0.199 | 0.362 |  | 0.834 | 0.124 | 0.040 |
| Model 2 | 0.631 | 0.353 | 0.652 | 0.061 |  |  |  |

SEP: Socioeconomic position; B-SEVLT: Brief-Spanish English Verbal Learning Test; WF: Word fluency; DSS: Digit symbol substitution; SIS: Six item screener; MCI: Mild cognitive impairment. Measures of childhood SEP: Parental education, maternal education, and paternal education (less than high school vs. high school or above), adult SEP (low vs. high SEP), socioeconomic mobility (reference: enduring high SEP)

**Table S2. Association between adult socioeconomic position with change in cognitive function and prevalent mild cognitive impairment, excluding participant’s education from the index of adult socioeconomic position**

| **Outcomes** | **Controlled Direct Effect^*^** | |
| --- | --- | --- |
|  | **Adult SEP** | **Adjusted p-value^£^** |
| **Change in cognitive function, β (95% CI)** |  |  |
| ∆Global cognition | -0.07 (-0.12, -0.02)^ⴕ^ | 0.005 |
| ∆B-SEVLT sum | -0.15 (-0.23, -0.07)^‡^ | 0.000 |
| ∆B-SEVLT recall | -0.12 (-0.21, -0.04)^ⴕ^ | 0.005 |
| ∆WF | -0.07 (-0.13, -0.01)^ⴕ^ | 0.037 |
| ∆DSS | -0.04 (-0.09, 0.01) | 0.136 |
| ∆SIS | -0.13 (-0.22, -0.05)^ⴕ^ | 0.005 |
| **Prevalent MCI, OR (95% CI)** | 1.42 (1.03, 1.95)^ⴕ^ | 0.037 |

SEP: Socioeconomic position; MCI: Mild cognitive impairment; CI: Confidence interval; B-SEVLT: Brief-Spanish English Verbal Learning Test; WF: Word fluency; DSS: Digit symbol substitution; SIS: Six item screener.

^*^The outcome regression models were weighted with the stabilized inverse probability weights to adjust for the set of confounders defined for adult SEP. We also included directly in the outcome regression model the relevant baseline confounders (year of birth, sex, place of birth, and Hispanic/Latino background) used to estimate the numerator of the inverse probability weights. The β coefficients and OR show the controlled direct effect of adult SEP on cognitive change and MCI. **^£^**p-value adjusted for false discovery rate using the Benjamini-Hochberg correction method

**Table S3. Association between socioeconomic mobility with change in cognitive function and prevalent mild cognitive impairment, using parental education and participant’s education as proxy for childhood and adult SEP, respectively**

| **Outcome** | **Upward mobility^*^** | | **Downward mobility^*^** | | **Enduring low SEP^*^** | |
| --- | --- | --- | --- | --- | --- | --- |
|  | **Association** | **Adjusted p-value^£^** | **Association** | **Adjusted p-value^£^** | **Association** | **Adjusted p-value^£^** |
| **Change in cognitive function, β (95% CI)** | | | | | | |
| ∆Global cognition | 0.01 (-0.05, 0.06) | 0.943 | -0.13 (-0.24, -0.02)^ⴕ^ | 0.049 | -0.12 (-0.18, -0.06)^ⴕ^ | 0.000 |
| ∆B-SEVLT sum | 0.04 (-0.04, 0.13) | 0.633 | -0.13 (-0.31, -0.06)^ⴕ^ | 0.205 | -0.12 (-0.22, -0.03)^ⴕ^ | 0.010 |
| ∆B-SEVLT recall | -0.00 (-0.09, 0.08) | 0.943 | -0.10 (-0.30, 0.09) | 0.307 | -0.12 (-0.21, -0.03)^ⴕ^ | 0.009 |
| ∆WF | -0.00 (-0.08, 0.07) | 0.943 | -0.22 (-0.35, -0.08)^ⴕ^ | 0.003 | -0.22 (-0.27, -0.14)^‡^ | 0.000 |
| ∆DSS | -0.06 (-0.12, -0.01)^ⴕ^ | 0.210 | -0.23 (-0.32, -0.13)^‡^ | 0.000 | -0.20 (-0.27, -0.14)^‡^ | 0.000 |
| ∆SIS | -0.09 (-0.18, 0.01) | 0.245 | -0.12 (-0.28, 0.03) | 0.166 | -0.16 (-0.26, -0.06)^ⴕ^ | 0.002 |
| **Prevalent MCI, OR (95% CI)** | 1.17 (0.78, 1.75) | 0.633 | 1.58 (0.89, 2.86) | 0.633 | 1.33 (0.91, 1.97) | 0.084 |

MCI: Mild cognitive impairment; SEP: Socioeconomic position; CI: Confidence interval; B-SEVLT: Brief-Spanish English Verbal Learning Test; WF: Word fluency; DSS: Digit symbol substitution; SIS: Six item screener.

^*^The outcome regression models were weighted with the stabilized inverse probability weights to adjust for the set of covariates defined for socioeconomic mobility. We also included directly in the outcome regression models the baseline confounders included to estimate the numerator of the inverse probability weights for socioeconomic mobility (year of birth, sex, place of birth, and Hispanic/Latino background). Socioeconomic mobility (reference: Enduring high SEP: high childhood and high adult SEP), upward mobility: low childhood to high adult SEP, downward mobility: high childhood to low adult SEP, enduring low SEP: low childhood and low adult SEP. **^£^**p-value adjusted for false discovery rate using the Benjamini-Hochberg correction method
